# The Role of Cardiac Troponins in Postmortem Diagnosis of Myocardial Ischemia: A Systematic Review

**DOI:** 10.3390/ijms26010105

**Published:** 2024-12-26

**Authors:** Matteo Antonio Sacco, Saverio Gualtieri, Gioele Grimaldi, Maria Daniela Monterossi, Valerio Riccardo Aquila, Alessandro Pasquale Tarallo, Maria Cristina Verrina, Francesco Ranno, Santo Gratteri, Isabella Aquila

**Affiliations:** Institute of Legal Medicine, Department of Medical and Surgical Sciences, “Magna Graecia” University, 88100 Catanzaro, Italy; matteoantoniosacco@gmail.com (M.A.S.); saverio.gualtieri@studenti.unicz.it (S.G.); gioele.grimaldi@studenti.unicz.it (G.G.); mariadaniela.monterossi@studenti.unicz.it (M.D.M.); valerioriccardo.aquila@studenti.unicz.it (V.R.A.); alessandropasquale.tarallo@studenti.unicz.it (A.P.T.); mariacristina.verrina@studenti.unicz.it (M.C.V.); francesco.ranno@studenti.unicz.it (F.R.); gratteri@unicz.it (S.G.)

**Keywords:** cardiac troponins, cTnI, cTnT, postmortem, myocardial ischemia, forensic pathology, postmortem interval, diagnostic biomarkers, sudden cardiac death, autopsy

## Abstract

Postmortem diagnosis of myocardial ischemia remains a challenge in forensic pathology, as traditional methods like autopsy and histology may not always provide conclusive results. Cardiac troponins, specifically cTnI and cTnT, are well-established biomarkers for myocardial injury in living patients, but their role in postmortem ischemia diagnosis is still under investigation. This systematic review aims to evaluate the role of troponins in diagnosing myocardial ischemia in postmortem cases, focusing on the diagnostic accuracy, sample types, and the influence of the postmortem interval (PMI). A systematic search on PubMed NCBI was conducted to identify studies published between 2000 and 2024 that evaluated the use of cTnI and cTnT in postmortem myocardial ischemia diagnosis. The studies were assessed for their sample types, methods of troponin quantification, sensitivity, specificity, and the impact of PMI on the stability of troponin levels. The review included 13 studies that utilized various sample types, including serum, femoral blood, and pericardial fluid. cTnT was found to be more reliable than cTnI, particularly in pericardial fluid, with shorter PMIs (typically under 48 h) showing higher sensitivity and specificity for myocardial ischemia. Pericardial fluid provided the most consistent results, followed by serum and femoral blood. Studies also highlighted that longer PMIs negatively affected the reliability of troponin measurements due to postmortem degradation. Cardiac troponins, especially cTnT, are valuable biomarkers for diagnosing myocardial ischemia postmortem, particularly when measured in pericardial fluid and within a short PMI. The use of multimarker strategies and the development of standardized cut-off values are needed to improve the accuracy of troponin measurements in forensic pathology. Future research should focus on standardizing diagnostic thresholds, improving assay sensitivity, and exploring new sample types and imaging techniques to enhance postmortem cardiovascular diagnostics.

## 1. Introduction

**Figure 1:**
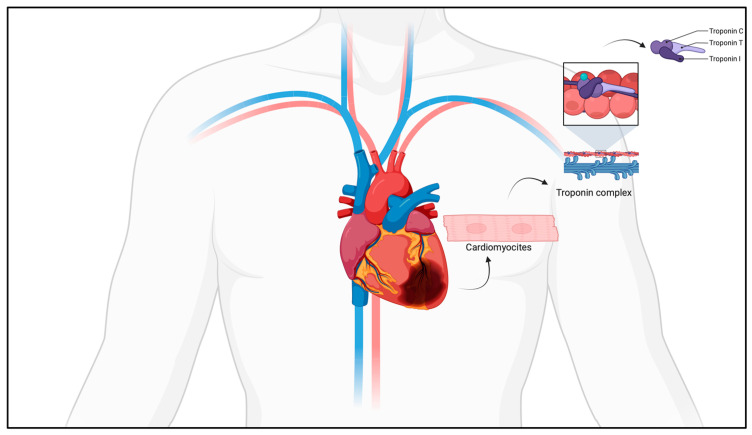
Schematic representation of troponin complex.

Myocardial ischemia is one of the leading causes of death worldwide, and its timely diagnosis is critical for both clinical treatment and forensic investigations. However, diagnosing myocardial ischemia postmortem presents a unique set of challenges, as the clinical signs typically observed during life may no longer be present after death. Traditional methods of postmortem diagnosis, such as autopsy and histological examination, can often be insufficient or inconclusive in detecting myocardial ischemia, particularly in cases where the ischemic event occurred shortly before death or when the body has undergone significant postmortem changes [1]. Cardiac troponins, specifically troponin I (cTnI) and troponin T (cTnT), have long been recognized as highly sensitive and specific biomarkers for diagnosing myocardial injury in living patients [2]. Cardiac troponins, specifically cardiac troponin I (cTnI) and cardiac troponin T (cTnT), are pivotal components in the regulation of cardiac muscle contraction. Troponins are a group of regulatory proteins that play a central role in the contraction of striated muscles, including the cardiac muscle. They are composed of three subunits—troponin C, troponin I, and troponin T—each with a distinct function that contributes to the regulation of muscle contraction. In the heart, troponin C serves as the binding site for calcium ions, which are released during the depolarization of cardiac muscle cells. This calcium binding triggers a conformational change in the troponin complex that allows the contractile machinery to function. Troponin I, on the other hand, acts as an inhibitory protein. In the absence of calcium, it prevents the interaction between actin and myosin, thereby inhibiting muscle contraction. When calcium binds to troponin C, this inhibition is lifted, enabling actin-myosin cross-bridge formation and initiating muscle contraction. Troponin T ensures the connection of the troponin complex to tropomyosin, a protein that stabilizes the filament system and coordinates the movement required for contraction (Figure 1).

Troponins are particularly important in the context of cardiac health because of their role as biomarkers for myocardial injury. Cardiac-specific isoforms of troponin I (cTnI) and troponin T (cTnT) are released into the bloodstream when cardiac myocytes are damaged, such as during acute myocardial infarction (AMI). The detection of elevated levels of cardiac troponins in blood tests is a cornerstone of modern diagnostics, providing a sensitive and specific measure of heart muscle damage. This makes troponins invaluable tools in clinical practice for the early detection and management of cardiac events. The specific functions of cTnI and cTnT render them highly sensitive markers for myocardial injury, as they are released into the bloodstream following cardiac muscle damage. This release mechanism is central to their utility in both clinical and postmortem settings, providing insight into the extent of myocardial ischemia [3].

While both cTnI and cTnT are integral to the diagnosis of myocardial injury, their diagnostic utility differs notably. Troponin T has been predominantly utilized in the clinical diagnosis of acute myocardial injury due to its stable release pattern and greater specificity in detecting myocardial necrosis. In contrast, cTnI is often preferred in certain clinical situations due to its unique amino acid sequence, which minimizes cross-reactivity with skeletal muscle, thus offering a more specific indication of cardiac muscle damage. However, in the postmortem context, both biomarkers are employed to ascertain cardiac death, and ongoing research seeks to clarify their respective diagnostic roles and effectiveness. The subtle differences in their diagnostic profiles highlight the importance of selecting the appropriate biomarker based on the clinical or forensic scenario.

These proteins are released into the bloodstream when myocardial cells are damaged, and their levels correlate closely with the extent of myocardial injury. As a result, troponins have become a cornerstone in diagnosing acute myocardial infarction and other heart-related conditions. However, their role in postmortem diagnostics, particularly in identifying myocardial ischemia, remains an area of active research [3,4]. Their ability to provide early detection of myocardial infarction is particularly crucial in forensic pathology, where timely and accurate diagnosis can significantly impact legal proceedings and family closure. The correlation between elevated troponin levels and the severity of ischemic myocardial highlights their diagnostic value, as higher concentrations often reflect more extensive damage to cardiac tissue. Additionally, the utility of troponins in postmortem analysis is increasingly recognized, offering critical insights into the cause of death and facilitating the understanding of cardiac pathophysiology in deceased individuals. This relevance in both living patients and postmortem cases underscores the versatility and essential nature of cardiac troponins in modern medical practice.

The use of troponins in postmortem settings is promising due to their high specificity for cardiac tissue, making them potentially invaluable for forensic pathologists in determining the cause of death [4]. However, the postmortem interval (PMI)—the time elapsed between death and the collection of the sample—plays a crucial role in the stability and detectability of these biomarkers. Troponins may degrade over time after death, which can affect their diagnostic accuracy, especially in cases where the PMI is long or where the body has undergone significant decomposition [5]. This review aims to explore the role of cardiac troponins, specifically cTnI and cTnT, in diagnosing myocardial ischemia in cadavers. We will examine how these biomarkers are used in postmortem forensic practice, their sensitivity and specificity in detecting ischemic damage to the heart, and the challenges posed by the PMI. By synthesizing the current literature, this review will offer a comprehensive understanding of the diagnostic value of cardiac troponins in postmortem ischemia and highlight areas where further research is needed to refine their use in forensic pathology. Ultimately, the goal is to assess whether cardiac troponins can be reliably employed as diagnostic tools for myocardial ischemia in cadavers, offering insights into their potential for improving forensic diagnoses of heart-related deaths.

## 2. Results

A total of 89 papers were obtained from the search. In particular, 25 were selected based on the abstracts. A total of 20 studies were included in this systematic review, evaluating the role of cardiac troponins (cTnI and cTnT) in diagnosing myocardial ischemia postmortem [1,6,7,8,9,10,11,12,13,14,15,16,17,18]. The studies varied in terms of sample types, troponin types measured, and the postmortem interval (PMI), which plays a significant role in the diagnostic accuracy of troponin measurements.

### 2.1. Study Characteristics

The studies included in this review used different sample types, including serum, femoral blood, pericardial fluid, and myocardial tissue. Among these, serum and femoral blood were the most commonly used, with studies like Kutlu E. et al. (2023) [1] and Moridi M. et al. (2022) [6] focusing on femoral blood as a key sample for measuring hs-cTnT and cTnT, respectively. Other studies, such as Sapouna R. et al. (2013) [14], utilized pericardial fluid, which provided higher sensitivity for detecting cardiac damage due to its proximity to the heart.

### 2.2. Postmortem Interval (PMI) and Troponin Stability

The postmortem interval (PMI) varied across the studies, ranging from 12 h to 48 h. Shorter PMIs were generally associated with higher sensitivity and specificity for troponin detection. For example, Kutlu E. et al. (2023) [1], with a PMI of less than 48 h, reported elevated hs-cTnT levels in serum and achieved significant diagnostic accuracy in cases of sudden cardiac death (SCD) due to ischemic heart disease. Similarly, Moridi M. et al. (2022) [6] found that cTnT in femoral blood had acceptable sensitivity (86.7%) but lower specificity (67.3%) when the PMI was between 12 and 48 h. Longer PMIs, as in cases with PMIs exceeding 48 h, showed diminished reliability for troponin detection due to degradation over time. For instance, in Beausire T. et al. (2018) [11], where the median PMI was 49 h, caution was recommended in interpreting hs-cTnT levels due to potential postmortem changes that could affect biomarker stability.

### 2.3. Diagnostic Accuracy and Sensitivity

Most studies demonstrated that cTnT is a more reliable postmortem marker for diagnosing myocardial ischemia compared to cTnI, with higher sensitivity in detecting ischemic changes. In Zribi M. et al. (2021) [8], hs-cTnT levels in pericardial fluid showed the best sensitivity (75%) and specificity (64%) for diagnosing sudden cardiac death (SCD). Furthermore, Cao Z. et al. (2019) [9], through a meta-analysis of 13 studies, established that both cTnI and cTnT had elevated levels in cases of cardiac death, and they suggested specific cut-off values for forensic applications.

Studies such as Barberi C. et al. (2018) [12] and Vanhaebost J. et al. (2016) [13] also highlighted the importance of cTnT in postmortem diagnosis, particularly in pericardial fluid and femoral blood. However, they noted the challenge of establishing consistent cut-off values, as troponin levels may vary depending on factors such as PMI, sample type, and the cause of death (Table 1).

## 3. Materials and Methods

A systematic search was conducted to identify relevant studies on the role of cardiac troponins (cTnI and cTnT) in the postmortem diagnosis of myocardial ischemia. The literature search included PubMed NCBI, which was selected for its extensive coverage of both medical and forensic literature. PRISMA guidelines were followed. The search terms used in this process included combinations of keywords such as “cardiac troponins” and “myocardial ischemia” and “autopsy”, ensuring a broad and inclusive capture of relevant articles. To ensure the quality and relevance of the studies included, specific inclusion and exclusion criteria were applied [19,20,21,22]. Studies were included if they were original research articles involving human cadavers or autopsy cases in which cardiac troponins (either cTnI or cTnT) were measured to diagnose myocardial ischemia. Only studies published in English were considered to ensure the review reflects the most current practices and techniques in forensic diagnostics. We excluded studies that did not directly measure troponins in a postmortem context or those that focused on non-human studies. Letters, editorials, and studies with insufficient data on the postmortem interval (PMI) were also excluded.

The study selection process was performed in two stages. First, titles and abstracts were screened for relevance. Articles that met the inclusion criteria were then subjected to full-text review. Disagreements between the reviewers were resolved through discussion or consultation with a third reviewer. Once the studies were selected, relevant data were extracted systematically using a standardized form to maintain consistency. Data points included study characteristics (authors, year of publication), sample size, type of troponin measured, assay methods, sample types (serum, femoral blood, pericardial fluid), and postmortem interval (PMI). Additionally, diagnostic outcomes such as the sensitivity, specificity, and accuracy of troponins in diagnosing myocardial ischemia were extracted. The quality of the included studies was assessed using the Newcastle-Ottawa Scale (NOS), a tool widely used for assessing the methodological quality of cohort studies. This scale evaluates studies based on three key categories: selection, comparability, and outcome. Each study was assigned a score ranging from 0 to 9, with higher scores reflecting better quality. Studies with scores below 5 were considered at high risk of bias, and a sensitivity analysis was conducted to determine the impact of these studies on the overall conclusions of the review.

**Figure 2:**
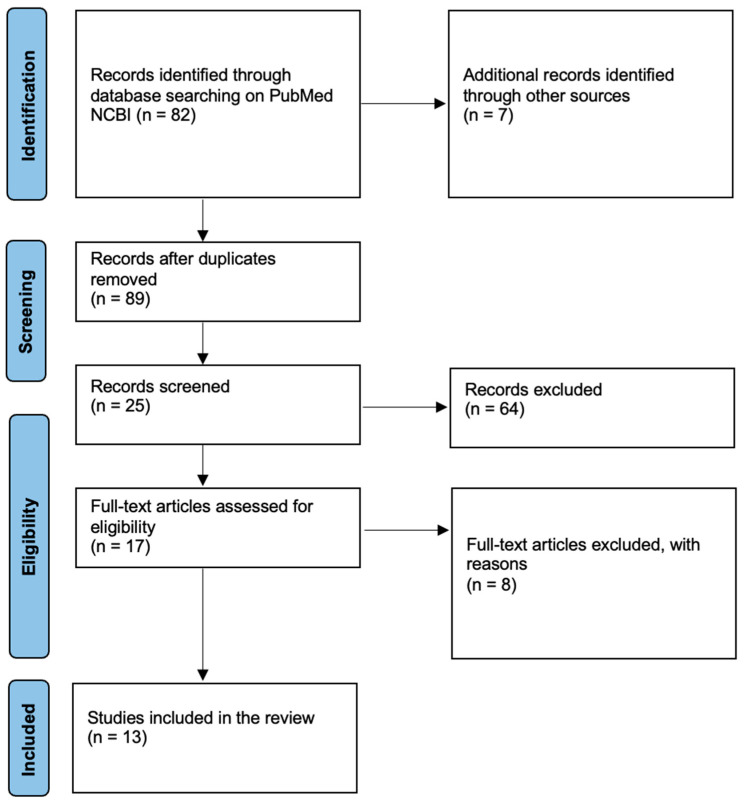
Searching method using PRISMA flowchart.

Given the variability in study designs, methodologies, and sample types, a meta-analysis was not feasible. Instead, a qualitative synthesis was performed. The studies were grouped according to factors such as the type of sample used (e.g., serum, femoral blood, pericardial fluid) and the troponin type measured (cTnI or cTnT). The results were then analyzed narratively, highlighting trends in diagnostic performance, particularly focusing on the sensitivity and specificity of cardiac troponins in diagnosing myocardial ischemia in postmortem cases. Special attention was given to the impact of the PMI on the stability and reliability of troponin measurements, as the postmortem interval is known to influence the degradation of biomarkers (Figure 2). 

## 4. Discussion

This systematic review has highlighted the significant potential of cardiac troponins (cTnI and cTnT) as postmortem biomarkers for diagnosing myocardial ischemia [6,7,8,9,10,11,12,13,14,15,16,17,18].

### 4.1. Diagnostic Techniques for Detecting Myocardial Ischemia After Death

The histological examination of cardiac tissues serves as a cornerstone in the diagnosis of myocardial ischemia after death. Through this technique, forensic pathologists can scrutinize the structural changes in heart tissues that are indicative of ischemic events. The presence of coagulative necrosis, contraction band necrosis, and inflammatory cell infiltration are some of the histopathological features that suggest myocardial ischemia. However, distinguishing these features from postmortem autolytic changes poses a significant challenge to forensic experts. Advanced staining techniques and microscopic analysis can aid in differentiating ischemic lesions from postmortem artifacts, thereby enhancing the accuracy of forensic examinations. Chemical analysis of myocardial tissue samples is another pivotal diagnostic technique employed to detect myocardial ischemia postmortem. This method involves the evaluation of specific biochemical markers that are released during cardiac injury. Among these markers, cardiac troponins, particularly Troponin I (cTnI) and Troponin T (cTnT), have gained prominence. These proteins are integral to the contractile mechanism of cardiac muscles and are released into the bloodstream during myocardial damage. Postmortem biochemical analysis of these troponins provides significant insights into cardiac death, especially in cases of acute myocardial infarction (AMI) [5,19,20,21,22].

The mechanisms leading to myocardial ischemia after death involve several complex processes. After death, the cessation of blood flow and oxygen delivery halts cellular metabolism, leading to an accumulation of metabolic waste products and a decrease in pH within the myocardial tissue [23]. This biochemical environment can mimic features of ischemia seen in living patients, but without the accompanying cellular responses like inflammation. Additionally, postmortem changes such as autolysis and putrefaction can further complicate the assessment of ischemic damage. Therefore, the use of cardiac troponins as biomarkers is particularly valuable, as they help to indicate the presence and extent of ischemic injury even in the absence of cellular responses. Troponin levels measured postmortem have been shown to correlate with the severity of ischemic damage, providing critical information for forensic investigations

The results from the included studies suggest that cTnT, particularly in pericardial fluid, is a more reliable marker for myocardial ischemia when compared to cTnI. However, the diagnostic utility of troponins is influenced by several factors, most notably the postmortem interval (PMI), sample type, and the methods used to measure these biomarkers. The PMI plays a critical role in the stability and reliability of troponin measurements. Shorter PMIs, particularly those under 48 h, were consistently associated with higher sensitivity and specificity for detecting myocardial ischemia. This is likely because troponins remain more stable in the early postmortem period, making them more detectable and reliable markers for ischemic heart disease. As the PMI increases, however, troponin levels tend to degrade due to enzymatic breakdown and other postmortem changes, leading to a decrease in diagnostic accuracy. This finding suggests that troponins are most useful in cases where the PMI is relatively short, and their utility diminishes with longer intervals after death.

The sample type used for troponin measurement is another important factor influencing diagnostic performance [6,7,8,9,10,11,12,13,14,15,16,17,18]. Pericardial fluid, in particular, emerged as the most reliable sample for detecting troponin levels in postmortem cases of myocardial ischemia. Its proximity to the heart likely contributes to its higher sensitivity, as it reflects the extent of myocardial damage more accurately than other bodily fluids. Serum and femoral blood, while commonly used, showed more variability in results. Serum samples, in particular, can be prone to postmortem degradation, affecting the reliability of the troponin measurements. Femoral blood, while providing useful data, is less specific to cardiac injury compared to pericardial fluid. The diagnostic performance of cTnT was generally superior to cTnI, especially in pericardial fluid. cTnT appears to remain detectable for a longer period after death, which may explain its higher diagnostic accuracy, particularly when the PMI is within a shorter time frame. cTnI, while useful, showed more variability in its postmortem performance and was less reliable in cases with longer PMIs.

### 4.2. Challenges and Limitations

Despite the promising findings, several challenges remain in the use of troponins for postmortem diagnosis. One major limitation is the lack of standardized cut-off values for both cTnI and cTnT in forensic settings. While some studies have proposed specific thresholds for cTnT, these values vary significantly depending on factors such as PMI, sample type, and the methodology used [6,7,8,9,10,11,12,13,14,15,16,17,18]. The establishment of standardized cut-off values is crucial for improving the consistency and reliability of troponin measurements in forensic pathology. Another challenge is the clinical correlation required for the accurate interpretation of troponin levels. Elevated troponin levels may be indicative of myocardial ischemia, but they can also be elevated in cases of other cardiac conditions or systemic illnesses. Among cardiac conditions, elevated troponin levels can be observed in myocarditis, where inflammation of the myocardium causes myocardial injury, leading to troponin release. Additionally, heart failure, particularly acute decompensated heart failure, is associated with elevated troponins due to increased myocardial wall stress and subsequent cardiomyocyte damage. Other cardiac causes include cardiomyopathies (e.g., hypertrophic cardiomyopathy, Takotsubo cardiomyopathy), severe arrhythmias such as ventricular tachycardia or atrial fibrillation with rapid ventricular response, and valvular heart disease, where volume or pressure overload can result in myocardial injury.

In terms of systemic illnesses, elevated troponin levels are commonly seen in conditions causing systemic inflammation, hypoxia, or hemodynamic stress. For example, sepsis and systemic inflammatory response syndrome (SIRS) can cause troponin elevation due to microvascular injury, hypoperfusion, and increased myocardial oxygen demand. Similarly, pulmonary embolism can elevate troponins as a result of right ventricular strain and ischemia. Chronic kidney disease (CKD) is another systemic condition associated with elevated troponins, likely due to reduced clearance and chronic myocardial injury. Additionally, stroke and other forms of acute neurological injury, such as subarachnoid hemorrhage, can lead to troponin release due to neurogenic cardiac injury, possibly mediated by catecholamine surges. Finally, conditions involving significant hypoxia, such as severe anemia or respiratory failure, may also cause myocardial injury and subsequent troponin elevation. Therefore, forensic pathologists must interpret troponin results in conjunction with other postmortem findings, such as histology, clinical history, and other diagnostic markers, to reach a definitive conclusion. The detection and characterization of troponin fragments have indeed proven valuable in clinical settings, as their degradation patterns reflect distinct processes of myocardial injury. For instance, strenuous exercise-induced elevations tend to involve intact troponins or specific degradation fragments, whereas ischemic injury associated with myocardial infarction typically results in more extensive proteolytic breakdown of troponins. In the postmortem context, the detection of troponin fragments could offer a novel and valuable tool for discriminating between different causes of myocardial injury. Given that postmortem conditions are subject to protein degradation and redistribution, studying the specific patterns of cTnI and cTnT fragmentation might enhance the ability to differentiate between ischemic events occurring antemortem and non-specific postmortem release of troponins. The role of proteolytic degradation, influenced by both ischemic conditions and postmortem autolysis, remains an area of active investigation. Future studies examining the stability and profiles of cTnI and cTnT fragments in postmortem samples could provide further clarity and strengthen their potential as biomarkers for myocardial ischemia in forensic pathology. At present, the use of troponin fragments in the postmortem setting is limited by a lack of standardized protocols for their detection and interpretation. However, given their potential to distinguish between ischemic and non-ischemic causes of troponin release, further research on the degradation kinetics of cTnI and cTnT fragments under postmortem conditions is warranted. This would not only improve diagnostic accuracy but also address some of the existing challenges associated with postmortem biochemistry.

### 4.3. Implications for Forensic Practice

While the use of cardiac troponins in postmortem diagnostics shows considerable promise, there are several avenues for future research that could further enhance their utility and accuracy in forensic pathology. One of the most pressing needs is the standardization of cut-off values for both cTnI and cTnT. Although some studies have suggested thresholds for these biomarkers, there is no universally accepted reference range for postmortem cases. Establishing standardized cut-off values would greatly improve the consistency and reliability of troponin measurements in forensic settings, allowing for more accurate differentiation between myocardial ischemia and other causes of death. In their study of 175 forensic cases, Hernandez-Romero et al. established that the cTnI ratio was significantly associated with AMI deaths, outperforming serum cTnI levels alone in diagnostic accuracy [24]. Specifically, they identified a cut-off ratio of 4.09, suggesting that ratios above this threshold are indicative of AMI. Importantly, their findings emphasize that this ratio can serve as an independent predictor of AMI-related death after adjusting for confounding factors such as age and postmortem interval. This is particularly relevant in cases where histological or macroscopic findings are inconclusive, a common challenge in sudden cardiac death investigations. The implications of their findings are profound for postmortem diagnostics. While pericardial fluid cTnI alone has diagnostic value, the pericardial fluid-to-serum ratio minimizes variability caused by postmortem redistribution and serum autolysis, enhancing the reliability of biochemical analysis. This approach could be highly beneficial for distinguishing myocardial ischemia from other causes of sudden death, such as trauma, asphyxia, or natural deaths. Further investigation into the impact of the postmortem interval (PMI) on troponin stability is also necessary. In addition, the potential for combining troponins with other biomarkers should be explored. A multimarker approach could enhance diagnostic accuracy by providing a more comprehensive understanding of the cause of death. Combining troponins with other postmortem markers such as NT-proBNP or creatine kinase MB (CK-MB) could improve the ability to distinguish between ischemic heart disease and other cardiac pathologies [23]. Furthermore, future studies could investigate the use of high-sensitivity assays for cTnI and cTnT, as these assays have the potential to detect even low levels of troponins, which could be crucial in cases with subtle myocardial injury.

Another promising area for research is the exploration of new sample types for troponin measurement. While pericardial fluid has shown superior diagnostic accuracy, its collection can be challenging in some cases. Investigating the use of alternative sample types, such as myocardial tissue, cerebrospinal fluid (CSF), or even saliva, could open up new opportunities for postmortem diagnostics. Lastly, the integration of advanced imaging techniques, such as postmortem CT-angiography or MRI, with biomarker analysis could significantly improve the diagnostic process. Combining troponins with non-invasive imaging could help identify ischemic myocardial damage more accurately, particularly in cases where autopsy findings are inconclusive or where traditional methods of diagnosis are insufficient. The use of multimodal diagnostic strategies could set a new standard for postmortem cardiovascular investigation, enhancing the ability to determine the true cause of death. Forensic pathologists must carefully consider these factors when utilizing troponins to diagnose.

## 5. Conclusions

In conclusion, cardiac troponins, especially cTnT, are promising biomarkers for diagnosing myocardial ischemia postmortem, particularly in the early postmortem period when the PMI is short. While pericardial fluid provides the most reliable results, serum and femoral blood can also be used effectively, though they may be less specific. The PMI plays a critical role in the accuracy of troponin measurements, and further research is needed to standardize cut-off values and optimize the use of troponins in forensic pathology. Standardizing diagnostic protocols and incorporating troponins into multimarker strategies could further improve postmortem diagnosis of myocardial ischemia and sudden cardiac death.

## Figures and Tables

**Table 1:** Results of systematic review of literature.

Author	Year	Title	Focus	Sample Type	Dosage/Quantification	Number of Cases	Troponin Type	Postmortem Interval (PMI)	Conclusion
Kutlu E. et al. [1]	2023	Significance of postmortem biomarkers and multimarker strategy in sudden cardiac death	Evaluation of hs-cTnT and other markers in postmortem serum for sudden cardiac death due to ischemic heart disease.	Femoral vein blood (serum)	hs-cTnT: mean 1225.6 ng/L, cut-off 36.22 ng/L; NT-proBNP: mean 932.3 pg/mL, cut-off 136.5 pg/mL.	59	cTnT	<48 h	hs-cTnT and NT-proBNP were significantly elevated in SCD cases. Multimarker strategy improves diagnostic accuracy.
Moridi M. et al. [6]	2022	Cardiac troponin T as a postmortem biomarker for acute myocardial infarction	Evaluation of postmortem cTnT levels in femoral blood and pericardial fluid for diagnosing AMI.	Femoral vein blood and pericardial fluid	Femoral blood: cut-off at 56 ng/L; Pericardial fluid: cut-off at 206,000 ng/L.	64	cTnT	12–48 h	Serum cTnT showed acceptable sensitivity (86.7%) but limited specificity (67.3%) for AMI diagnosis. Pericardial fluid had lower specificity.
Omalu B. et al. [7]	2021	Autopsy Cardiac Troponin I Plasma Levels Can Be Elevated in Myocardial Infarction Type 3	Postmortem cTnI measurements for MI type 3 diagnosis.	Serum	cTnI: range from 0.31 to >4400 ng/mL	52	cTnI	Not reported	Elevated cTnI observed in 90% of MI type 3 cases at autopsy.
Zribi M. et al. [8]	2021	Diagnostic value of high-sensitivity troponin T in postmortem diagnosis of sudden cardiac death	Assessment the diagnostic value of postmortem dosage of Hs-TnT in the diagnosis of sudden cardiac death.	Pericardial fluid, cardial blood e peripheral blood	The pericardial fluid had the best sensibility (75%) and specificity (64%) with a cut-off level at 17.72 ng/m	80	cTnT	12–24 h	Cardiac troponin T by a highly sensitive assay in pericardial fluid may be a powerful aid in the postmortem diagnosis of sudden cardiac deat
Cao Z. et al. [9]	2019	Diagnostic Roles of Postmortem cTn I and cTn T in Cardiac Death	Meta-analysis of postmortem cTn I and cTn T levels for cardiac death diagnosis.	Serum and pericardial fluid	cTn I in serum: 9.5 ng/mL (and in Pericadial fluid 86.2 ng/mL); cTn T in serum: 8.025 ng/mL	13 studies with hundreds of cases were analyzed	cTnI and cTnT	<48 h	Troponins elevated in cardiac death; thresholds established for forensic applications.
Buja L.M. et al. [10]	2019	Clinicopathological complexity in the application of the universal definition of myocardial infarction	Challenges of applying the Universal Definition of Myocardial Infarction in postmortem diagnostics.	Serum and myocardial tissue	Emphasizes importance of hs-cTn as a diagnostic marker.	5	hs-cTnT	not reported	Clinical correlation essential for reliable interpretation of troponin levels.
Beausire T. et al. [11]	2018	High-sensitive cardiac troponin hs-TnT levels in sudden deaths related to atherosclerotic coronary artery disease	Evaluation of hs-TnT serum levels in postmortem sudden deaths related to ischemic heart disease.	Serum	hs-TnT: Mean 1709.3 ng/L in cases, with cut-off thresholds at 12 ng/L, 2250 ng/L.	85	hs-cTnT	49 h mediana	A significant non-linear association between hs-TnT serum values and sudden deaths due to IHD; caution in interpretation due to postmortem alterations.
Barberi C. et al. [12]	2018	The use of cardiac troponin T (cTnT) in the postmortem diagnosis of acute myocardial infarction and sudden cardiac death: A systematic review	Reviews the use of cardiac troponin T (cTnT) in postmortem diagnosis, noting that it remains stable up to 48 h and is more concentrated in pericardial fluid. There is no consensus on the cut-off values for cTnT in forensic use	Pericardial fluid, cardial blood e peripheral blood and CSF	has not been easy to establish a common cut-off for the postmortem value of this marker	1923	cTnT	<48 h	cTnT in postmortem fluids adds information concerning the extent of the myocardial damage
Vanhaebost J. et al. [13]	2016	Diagnosis of myocardial ischemia combining multiphase postmortem CT-angiography, histology, and postmortem biochemistry	Correlation of MPMCTA findings with increased troponin levels and histological myocardial ischemia indicators.	Postmortem femoral blood	Troponin I: 1.21–10.16 µg/L; Troponin T: 32–98 ng/L	80	cTnI and cTnT	Not reported	Increased troponin levels systematically correlated with myocardial ischemia findings on MPMCTA.
Sapouna R. et al. [14]	2013	Diagnostic value of cardiac troponin I in postmortem diagnosis of myocardial infarction	Evaluation of cardiac troponin I (cTnI) as a postmortem marker for diagnosing myocardial infarction.	Pericardial fluid	cTnI: Mean concentration of 1067.03 mg/dL for MI cases.	89 (of which 28 cases were miocardial infarction)	cTnI	Not reported	Postmortem cTnI levels provide a reliable tool for diagnosing myocardial infarction in medicolegal autopsies.
Remmer S. et al. [15]	2013	Cardiac troponin T in forensic autopsy cases	Evaluation of cardiac Troponin T as biomarker in cardiovascular death	Femoral/iliac vein blood and pericardial fluid	serum: cut-off 0.6 ng/mL; pericardial fluid: cut-off 100 ng/mL	101	CtnT	8–141 with a median 34.5 h	cTnT levels may halp to differentiate cardiovascular death from other
Tomásková E., Vorel F. [16]	2010	Some possibilities in the diagnosis of early acute ischaemic changes in the heart muscle in sudden death	Evaluation of troponin I levels in postmortem diagnosis of early ischemic changes in the heart muscle.	Serum	cTnI: 0.13–2.80 µg/L	71	cTnI	12–36 h	Troponin I is a useful marker for detecting early ischemic changes in sudden cardiac death, especially in cases within the early PMI range.
Batalis N.I. et al. [17]	2010	The role of postmortem cardiac markers in the diagnosis of acute myocardial infarction	Evaluation of postmortem cardiac markers, including troponin I, in diagnosing acute myocardial infarction in forensic investigations.	Serum and pericardial fluid	not reported	10	cTnI	<24 h	Postmortem cTnI levels provide reliable evidence for diagnosing acute myocardial infarction, especially within the first 48 h of death.
Zhu B.-L. et al. [18]	2007	Postmortem cardiac troponin I and creatine kinase MB levels in the blood and pericardial fluid as markers of myocardial damage in medicolegal autopsy	Examines cardiac troponin I and creatine kinase MB, which increase after death and vary according to the cause of death.	Central and peripheral blood, pericardial fluid	cTnT: 0.03–50 ng/mL; CK-MB 0.3–150 ng/ml	234	CtnT and CK-MB	4–48 h	The research establishes that postmortem concentrations of cardiac troponin I and creatine kinase MB in blood and pericardial fluid serve as markers for myocardial injury and differ depending on the cause of death.

## Data Availability

Not applicable to this article as no datasets were generated.
